# A study of the effect of question feedback types on learning engagement in panoramic videos

**DOI:** 10.3389/fpsyg.2025.1321712

**Published:** 2025-02-27

**Authors:** Guan Huang, Haohua Zhang, Jingsheng Zeng, Wen Chen

**Affiliations:** ^1^College of Education, China West Normal University, Nanchong, China; ^2^Cyberspace Security Academy, Sichuan University, Chengdu, China

**Keywords:** panoramic video, question feedback, cognitive engagement, emotional engagement, behavior engagement

## Abstract

**Introduction:**

The immersive and interactive nature of panoramic video empowers learners with experiences that are infinitely close to the real environment and increases the use of imagination in learners’ knowledge acquisition. Studies have shown that embedding question feedback in traditional educational videos can effectively improve learning. However, little research has been conducted on embedding question feedback in panoramic videos to explore what types of question feedback effectively improve the dimensions of learners’ learning engagement and yield better learning experiences and learning effects.

**Methods:**

This study embedded questions with feedback within panoramic videos by categorizing feedback into two types: simple feedback and elaborated feedback. Using eye tracking, brainwave meters, and subjective questionnaires as measurement tools, this study investigated which type of question feedback embedded in panoramic videos improved various dimensions of learner engagement and academic performance. Participants (*n* = 91) were randomly assigned to the experimental group (simple feedback, elaborated feedback) or the control group (no feedback).

**Results:**

The results of the study showed that (1) the experimental group significantly improved in cognitive engagement, behavioral engagement, and emotional engagement compared to the control group. When the precision of feedback information was greater, the learner’s behavioral engagement was greater; however, the precision of feedback information did not significantly affect cognitive and emotional engagement. (2) When the feedback information was more detailed, the learners’ academic performance was better.

**Discussion:**

The findings of this study can support strategic recommendations for the design and application of panoramic videos.

## 1 Introduction

There has been great progress in the field of virtual reality (VR) technology in recent years, and there is a growing demand for VR technology in various industries; the Horizon Report considers VR technology to be one of the key technologies for promoting informatization and intelligence in education (Lan et al., 2021). Panoramic video supported by VR technology brings new vitality to technology-enabled education and guides a new direction for educational innovation. The almost real learning environment that it creates can provide learners with an immersive feeling. Panoramic videos can be viewed or controlled in a low immersive manner on a desktop or in a highly immersive way with head-mounted-displays (HMDs) (Rosendahl and Wagner, 2023). However, due to the omnidirectional view of panoramic videos, learners usually get lost in the wide view and thus miss the learning content that they should pay attention to, leading to the possible problem of inattention while learning (Johnson, 2018) and thus making it difficult for this technology to significantly impact academic performance (Panchuk et al., 2018).

Embedding questions in traditional instructional videos is an important form of interaction that significantly impacts learning. For example, it can significantly improve learners’ academic performance (Rose et al., 2016) and effectively help learners avoid the tendency to wander off during the learning process (Szpunar et al., 2013). The design and application of embedded questions are also becoming increasingly widespread to improve the interactivity and teaching effectiveness of instructional videos (Callender and McDaniel, 2007). Feedback can be defined as information that is provided to learners about their performance or behavior (Hattie and Timperley, 2007). This study defines question feedback as the presetting of questions based on panoramic video learning content, where the questions are embedded into the panoramic video and learners are provided with feedback information after they answer the questions.

Panoramic video provides a low-cost opportunity for video instruction (Roche et al., 2021) that extends the benefits of traditional video through immersion and multiview reflection (Rosendahl and Wagner, 2023). Therefore, the question of how to increase the interaction between learners and videos and improve learner engagement in an immersive learning environment such as a panoramic video by embedding question feedback deserves attention. The aims of this study are to investigate whether the presence or absence of feedback within panoramic videos embedded with questions has an impact on learners’ engagement and academic performance and to explore the impact of different types of feedback on learning engagement and academic performance. The findings of this study will provide guidance for the design and development of educational panoramic videos.

## 2 Literature review

### 2.1 The application of panoramic videos in education

Panoramic video is shot with an omni-directional camera, which allows the viewer to see the scene in an uninterrupted circle, as opposed to the fixed point of view of traditional 2D video (Meyer et al., 2019). Numerous studies have investigated the pedagogical applications of panoramic videos. For example, Araiza-Alba et al. (2020) compared three media (panoramic video, traditional video, and posters) for learning water safety skills and found that children learning through panoramic videos experience greater interest and enjoyment and that panoramic videos are more encouraging and engaging than traditional methods of learning. Ulrich et al. (2021) compared panoramic videos to traditional videos and found that panoramic videos can influence students’ emotional responses to the learning atmosphere in a positive way. Rupp et al. (2019) examined the effects of viewing a water safety video on a smartphone with four devices with different levels of immersion (a smartphone, Google Cardboard, Oculus Rift DK2, and Oculus CV1) to watch space-themed panoramic educational videos and found that the highly immersive experience of panoramic videos resulted in a positive learning experience, improved learning outcomes, and increased engagement and attention. Li et al. (2020) conducted a quasi-experimental study on the empathic accuracy of 68 teacher trainees by recording VR panoramic videos in a fixed camera position and found that VR panoramic videos were more effective at improving teacher trainees’ empathic accuracy than traditional videos. Although most studies on the learning effects of panoramic videos have shown positive effects, some studies have shown that panoramic videos distract students (Rupp et al., 2016), make them physically uncomfortable (Wilkerson et al., 2022), and ultimately fail to improve or even inhibit learning effects. Therefore, researchers have not yet reached a consensus on the effectiveness of panoramic videos in educational applications.

### 2.2 Question feedback types

Previous studies have investigated types of feedback on questions. Hsieh and O’Neil (2002) found that learners learning from videos embedded with questions learned better when elaborated feedback was provided than when simple feedback was provided. Levant et al. (2018) compared two types of delayed feedback (correct feedback and correct feedback with justification) provided to students after an exam in a computer-based testing system and found that the delayed feedback that provided not only correct feedback but also a rationale resulted in a greater increase in exam scores. Lin et al. (2013) linked instructional proxies to verbal feedback (simple and elaborated feedback) and showed that participants who learned using an animated proxy that provided detailed feedback scored significantly higher on a learning measure. Lang et al. (2022) investigated the effects of both emotional and detailed feedback on multimedia learning with an instructional agent and found that well-designed feedback increased intrinsic motivation and transfer scores but reduced the associated cognitive load. Lin (2011) found that among three types of static and dynamic visual materials—without questions, with questions, and with both questions and feedback—learning with questions and feedback was the best. Xie et al. (2021) used an eye-tracking experiment to examine the effects of pre-embedded questions and feedback design in instructional videos on learners’ attention allocation and learning performance and found that pre-embedded questions without feedback not only increased learners’ attention to the content but also improved their learning performance. According to the feedback principle in multimedia learning, novice students learn better with explanatory feedback than with corrective feedback alone (Mayer, 2014). Explanatory feedback provides learners with a principle-based explanation for why their answer was correct or incorrect, whereas corrective feedback merely informs learners that their answer was correct or incorrect (Mayer, 2014). Some scholars have also found that feedback is not positively effective for learning outcomes. For example, Kluger and DeNisi (1996) found that feedback interventions instead decrease performance, and Pashler et al. (2005) reported that timely or delayed feedback after a participant has answered correctly has little or no effect on learning outcomes. Jordan (2012) noted that feedback can be effective if learners can understand the content of the feedback. If the feedback provided can explain the errors made by learners rather than just providing answers, then it is more helpful in promoting learning.

### 2.3 Learning engagement

Learning engagement is the energy, flexibility, and positive emotions that an individual displays during the learning process and reflects the learner’s comprehension of the nature of learning and immersion in it (Wu and Zhang, 2018). Fredricks et al. (2004) argued that learning engagement is the emotional and behavioral engagement of students through the learning process and encompasses three independent dimensions: behavioral, emotional, and cognitive. Learning engagement has a determining influence on the effectiveness of active learning. In this study, learning engagement is the intrinsic and extrinsic behavioral state of learners through the learning process by actively participating in information exchange activities and by investing time and energy into deep thinking, which includes three dimensions: cognitive engagement, behavioral engagement, and emotional engagement.

As an important indicator for observing the learning process and measuring the quality of learning (Kim et al., 2021), learning engagement has a direct positive effect on learning outcomes (Hu et al., 2020). For example, Pascarella et al. (2010) found that learning engagement can positively impact academic performance, Pizzimenti and Axelson (2015) concluded that the level of students’ learning engagement can effectively predict their learning outcomes, and Liu (2022) determined that when the learning engagement of hearing-impaired students is greater, they are more willing to devote their time and energy to learning, which is conducive to improving academic performance. These studies show that learning engagement is an important factor that affects learning outcomes and deserves the attention of researchers.

Numerous studies have measured learning engagement. Junco (2012) used a questionnaire to study the impact of over two thousand school students’ use of social media on their learning engagement and found that indulgence in social media was detrimental to students’ learning engagement in the classroom. Zheng et al. (2021) developed a virtual simulation experimental teaching platform for situational English, used a questionnaire survey method, and found that enhancing the motivation of English language learners promoted their level of learning engagement. Cao et al. (2019) observed that the multimodal learning engagement recognition method is superior to the unimodal learning engagement recognition method. Using a multimodal data analysis approach, Wang et al. (2021) determined that learners’ cognitive engagement and behavioral engagement were greater in desktop VR learning environments than in online learning environments. Moreover, learning engagement is affected by the learning environment, and both directly intervening in the learning process and changing the learning environment can affect learner engagement (Lawson and Lawson, 2013).

### 2.4 The current study

Based on the above analysis, it is worth further verifying whether the impact of question feedback types on learning in panoramic videos is the same as that in traditional videos. This study focuses on exploring what types of feedback can enhance learners’ learning engagement and academic performance in a panoramic video environment while also comparing the impact of the presence or absence of feedback on learning engagement and academic performance. Therefore, the following research questions are proposed in this study:

1: What type of question feedback is embedded in panoramic videos to improve learning engagement and academic performance?
2: What impact does the presence or absence of feedback in panoramic videos with embedded questions have on learning engagement and academic performance?

## 3 Materials and methods

### 3.1 Participants

This study selected a total of 127 undergraduate students from M University, aged between 19 and 21 years old, with normal or corrected-to-normal vision. To eliminate the impact of prior knowledge on academic performance, participants who were relatively familiar with the experimental materials were excluded through a pre-test questionnaire. The remaining 102 individuals, who had little understanding of the relevant information in the experimental materials, were chosen as subjects and randomly divided into three groups.

### 3.2 Research hypotheses

This study developed a panoramic video that embeds questions and provides feedback. Referring to Shute’s (2008) classification of feedback types, question feedback types were divided into simple feedback and elaborated feedback. Simple feedback refers to providing learners with only correct or incorrect answers, while elaborated feedback indicates providing learners with not only correct or incorrect answers but also detailed explanations of the question. The research hypotheses of this study are as follows:

H1: Compared to simple feedback, elaborated feedback can better enhance learners’ learning engagement and academic performance.

H2: Compared to panoramic videos without feedback, providing feedback can enhance learners’ learning engagement and academic performance.

### 3.3 Materials and equipment

#### 3.3.1 Video material

**FIGURE 1:**
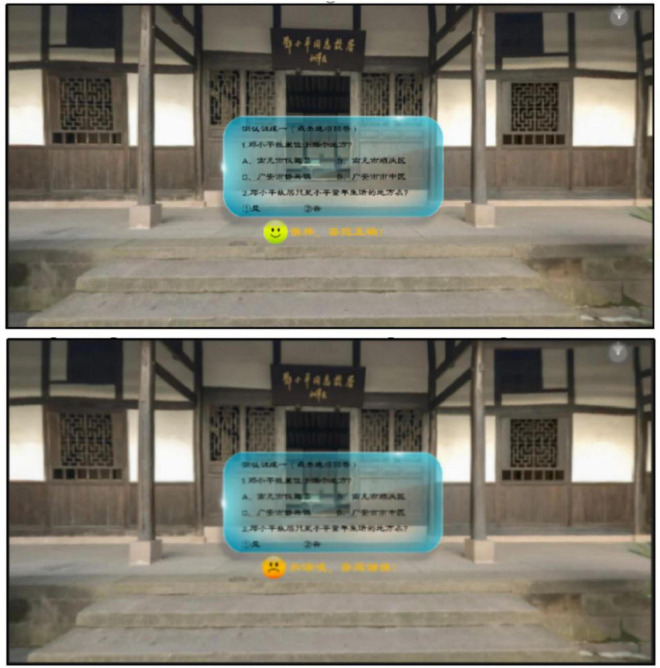
Example of simple feedback.

**FIGURE 2:**
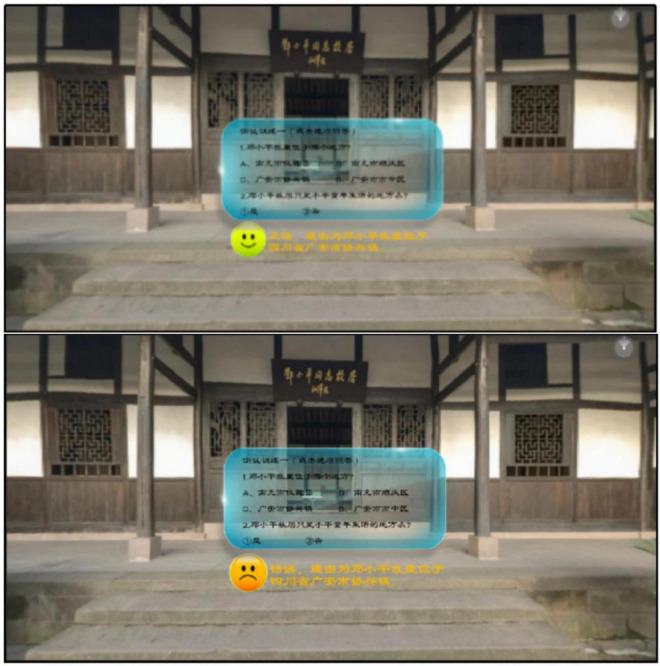
Example of elaborated feedback.

The panoramic video resources embedded with question feedback in this study are mainly developed in Unity 3D, and the learning material is a video about “Deng Xiaoping, the Great Man,” which shows the site of Deng Xiaoping’s former residence and exhibition hall, briefly explains Deng Xiaoping’s biography and experiences, and then explains the main contents of Deng Xiaoping’s theory of socialism with Chinese characteristics, the development strategy of socialist construction, and the reform and opening-up. The panoramic video discussed in this study was viewed with an HMD, thus increasing the immersion of the learners during the learning process and enabling them to turn their heads 360° to view the entire world as in real life. The experimental materials consisted of three versions: a no-feedback version, a simple-feedback version, and an elaborated-feedback version. The study standardized the simple feedback phrases as “Great, correct answer!” or “Go for it, wrong answer!” and used consistently “Correct, the question is about the core knowledge and explanation” or “Wrong, the question is about the core knowledge and explanation” for elaborated feedback. The content of the narration in the panoramic video and the embedded questions and feedback are shown in Appendix Table 1. An example of simple question feedback embedded in question one is shown in Figure 1, and an example of elaborated question feedback is shown in Figure 2.

#### 3.3.2 Measurement methods and tools

Measurement of prior knowledge and its instruments: All pre-knowledge test questions were related to the main events of Comrade Deng Xiaoping’s great and glorious life, with a total of 16 single-choice questions.

The tools used to measure learning engagement are shown in Table 1.

**TABLE 1:** Measurement tools for learning engagement.

Dimension of measure-ment	Measure-ment tools	Measure-ment indicators	References
Cognitive engagement	Questionnaire	Questionnaire subjective data	Fredricks et al., 2005 Sun, 2014
	Eye-tracking device	Total gaze duration	–
		Total number of looks	
	Brain wave	Concentration	Smith and Kosslyn, 2006
Behavioral engagement	Questionnaire	Questionnaire subjective data	Skinner et al., 2008
	Brain wave	Duration of study	Li et al., 2018
Emotional engagement	Questionnaire	Questionnaire subjective data	Watson et al., 1988
	Brain wave	Relaxation	Kumar et al., 2014

In this study, an eye-movement version of an HTC VIVE Pro EYE model helmet was used, and the eye-movement data were recorded and collected in Tobii Pro VR Analytics; the computer equipment used was HP Z620 as the running workstation for the running of the software required for the experiment and the display of the panoramic video learning materials. A BrainLink-Lite portable brainwave meter from Hongli Technology was used to monitor and record the subjects’ brainwaves and other mental state data in real time.

Knowledge post-test questionnaire: The post-test questionnaire was compiled by combining the pre-test questionnaire and the knowledge points in the study materials of “Deng Xiaoping, the Great Man,” which focused on the main content of Deng Xiaoping’s theory of socialism with Chinese characteristics, the international situation at the time, the basis for the formation of Deng Xiaoping’s theories, the significance of Deng Xiaoping’s theories during the revolutionary period, the content and significance of the basic line of the primary stage of socialism, and the main content of the Reform and Opening Up. The questionnaire contained 16 multiple-choice questions.

#### 3.3.3 Data collection methods

The eye movement data in this study were recorded and collected in Tobii Pro VR Analytics, a plug-in that provides metrics such as total gaze time, total number of gazes, first gaze time, interaction time, and number of interactions. The study used the data recording and export function provided by the plug-in to select the relevant eye movement indicators to be exported after the data recording was completed, and the eye movement behavior indicators required for export in this experiment were mainly the data such as total gaze time and total number of gaze times.

##### 3.3.3.1 Eye movement data recording

**FIGURE 3:**
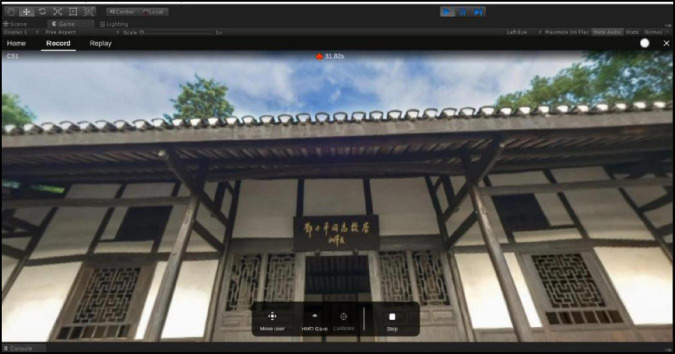
Eye movement data recording interface.

To start recording eye movement data, the VR headset needs to be activated, which can be done in the Home tab. When everything is ready, in the main recording interface, the “New Recording” button in the “Recording” control bar is used to record the data, and in the state of data recording, the learning process can be viewed in real time through the VR view provided by SteamVR. In the state of data recording (as shown in Figure 3), you can view the learner’s learning process in real time through the VR view provided by SteamVR.

##### 3.3.3.2 Exporting eye movement data

**FIGURE 4:**
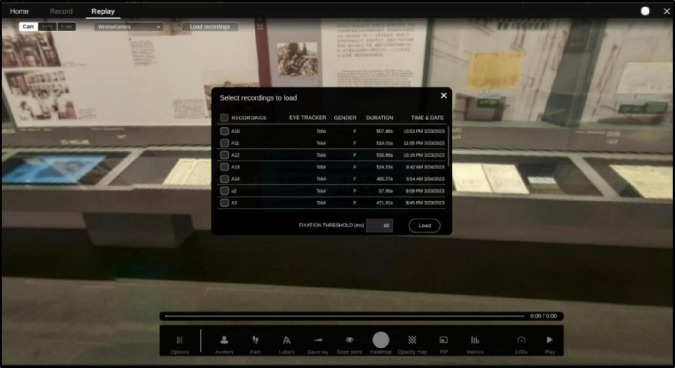
Eye movement data loading interface.

Finally, the eye movement data were exported after the experiment was completed. Enter the replay mode of Tobii Pro VR Analytics, select “Record” in the eye movement data export menu bar to bring up the eye movement data recording interface (Figure 4), check the experimental record, and then select “Record” in the eye movement data export control bar at the bottom of the page to export the data. Select “Record” in the eye movement data export menu bar to bring up the eye movement data recording interface.

##### 3.3.3.3 Recording and exporting of brainwave data

**FIGURE 5:**
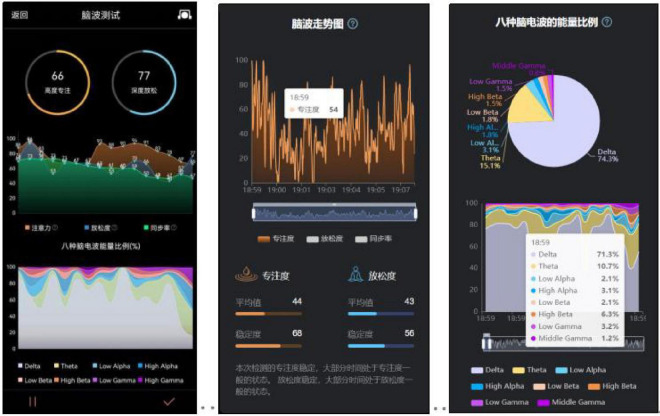
Brainwave data recording.

The brainwave data of this experiment are recorded and exported in the APP accompanying BrainLinkd, through which the trend of brainwave data and brainwave energy can be monitored in real time, Figure 5 is the trend of the brainwave after the completion of the data recording process, and the brainwave energy at a certain moment can also be viewed. Finally, select the brainwave data recording in APP to export and analyze the experimental data.

### 3.4 Experimental process

#### 3.4.1 Pre-testing

This experiment was conducted in the Virtual Reality Laboratory at the University of M. One week before the start of the experiment, all subjects were summoned to a computer room to randomly obtain a test serial number and complete the knowledge pre-test questionnaire. They then entered the VR training room to adapt to and use the VR equipment.

#### 3.4.2 Formal experiments

The experimental assistant led the subjects to wait for the experiment in the waiting room, the subjects entered the formal measurement laboratory and provided their demographic information, and the researcher informed the subjects about the experiment and the use of the study materials and precautions and waited for the subjects to familiarize themselves with the experimental environment before starting the formal experiment. The HTC helmet and brainwave meter were worn according to the subject’s actual condition, and the helmet was adjusted for comfort. It was confirmed that the subject could see the panoramic video, the subject was assisted in calibrating his or her eye movements through the five-point calibration method built into the HTC helmet, and the handle was given to the subject. Then, the researcher turned on the brainwave meter and recorded the eye movement data, and the subjects formally watched the video and learned the experimental material; during the video, they answered questions by clicking on the options using the handles.

#### 3.4.3 Post-testing

**FIGURE 6:**
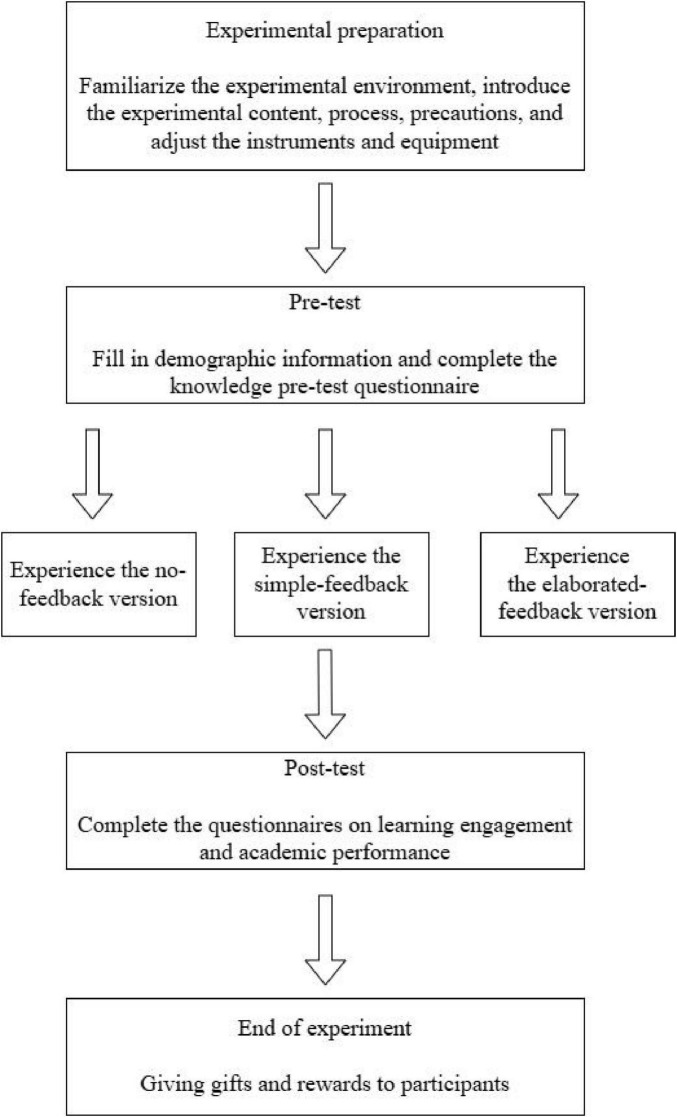
Experimental flow chart.

After the subjects completed the study, the researcher stopped recording the data, helped the subjects unlock the HTC helmet, and guided the subjects to sit in the corresponding area. The participants then completed the Learning Engagement Questionnaire and the Knowledge Post-test Questionnaire. The specific flow of the experiment is shown in Figure 6.

## 4 Results

This application study of panoramic video teaching resources embedded with question feedback mainly examined the impact of learning effects on learning engagement and academic performance, and experimental data with subjective and objective measurements were used to illustrate the results of the experiments in a comprehensive analysis. After eliminating the subject samples with an incomplete sampling of eye movement data or brain wave data, a total of 91 samples remained. In this study, descriptive statistics of learners’ prior knowledge were conducted and the results are shown in Table 2.

**TABLE 2:** Results of descriptive statistics of learners’ prior knowledge.

Data sources	Dimension of measurement	Question feedback	Sample size	Average value	Standard deviation
Questionnaire	Pre-testing	No feedback	30	7.57	1.77
		Simple feedback	30	7.47	1.81
		Elaborated feedback	31	7.58	2.03

In order to test whether the learners’ prior knowledge level is at the same level, the variance chi-square test and one-way ANOVA were conducted successively with the pre-test scores between the groups as the dependent variable, and the one-way ANOVA results showed that [F (2, 88) = 0.033, *P* = 0.967 > 0.05], which indicated that the difference between the total scores of the three groups’ pre-test scores did not reach the level of significance, and that the learners’ knowledge level of the material of this experiment converged to the same level prior to the acceptance of the experiment. The level of knowledge mastery of the material of this experiment tends to be the same, and prior knowledge does not affect the results of the experiment. The results of one-way ANOVA are shown in Table 3.

**TABLE 3:** One-way analysis of variance (ANOVA) results for learners’ prior knowledge.

Dependent variable	Sum of squares (between groups)	Mean square	F	Significance
Pre-testing	0.234	0.117	0.033	0.967

### 4.1 Learning engagement

#### 4.1.1 Cognitive engagement

Descriptive statistics on the subjective measures of the cognitive engagement questionnaire, concentration, total gaze time, and total number of gazes were obtained, and the statistical results are shown in Table 4.

**TABLE 4:** Results of the descriptive statistics of cognitive engagement.

Data sources	Dimension of measurement	Question feedback	Sample size	Average value	Standard deviation
Questionnaire	Cognitive engagement	No feedback	30	18.70	3.39
		Simple feedback	30	19.17	2.90
		Elaborated feedback	31	20.45	2.81
Brain wave instrument	Concentration	No feedback	30	44.88	3.69
		Simple feedback	30	48.45	3.76
		Elaborated feedback	31	47.33	4.97
Eye movement meter	Total gaze duration	No feedback	30	58.94	9.62
		Simple feedback	30	68.07	11.98
		Elaborated feedback	31	72.48	8.67
	Total number of looks	No feedback	30	256.27	36.80
		Simple feedback	30	306.70	48.52
		Elaborated feedback	31	311.13	45.74

To further examine whether there was a significant difference in the subjective judgments of cognitive engagement among the groups for the three types of feedback, a one-way analysis of variance (ANOVA) was conducted to analyze the subjective judgments of cognitive engagement among the three groups. The results showed that the differences in the subjective cognitive engagement judgments among the three groups reached a significant level [F = 2.716 (2,88), *P* = 0.072 < 0.1]. *Post hoc* multiple comparisons of subjective judgments of cognitive engagement using least significant difference (LSD) revealed that the difference in the subjective judgment values of cognitive engagement between the no-feedback group and the elaborated-feedback group was significant (*P* = 0.027 < 0.05), and the differences between the no-feedback group and the simple-feedback group (*P* = 0.554 > 0.05) and between the simple-feedback group and the elaborated-feedback group (*P* = 0.103 > 0.05) were not significant.

To further examine whether there was a significant difference among the groups of learners’ levels of concentration for the three types of feedback, a one-way ANOVA was conducted to analyze the learners’ levels of concentration of the three groups. The results showed that the differences in concentration among the three groups reached the level of significance (F = 5.699, *P* = 0.005 < 0.05). *Post hoc* multiple comparisons of the learners’ levels of concentration using LSD revealed that there was a significant difference in concentration between the no-feedback group and the simple-feedback group (*P* = 0.001 < 0.05) and between the no-feedback group and the elaborated-feedback group (*P* = 0.025 < 0.05) and that the difference between the simple-feedback group and the elaborated-feedback group was not significant (*P* = 0.299 > 0.05).

To further examine whether there were significant differences in the total gaze time and the total number of gazes among the groups for the three types of feedback, a one-way ANOVA was conducted on the total gaze time and the total number of gazes of the three groups. The results showed that the differences among the three groups in total gaze time reached a significant level [F = 14.003 (2,88), *P* = 0.000 < 0.05] and that the differences among the three groups in the number of gazes reached a significant level [F = 14.497 (2,88), *P* = 0.000 < 0.05]. The difference in total gaze time among the three groups reached a significant level [F = 14.497 (2,88), *P* = 0.000 < 0.05). *Post hoc* multiple comparisons of the total gaze time and total number of gazes using LSD revealed that in terms of total gaze time, the difference between the no-feedback group and the simple-feedback group was significant (*P* = 0.001 < 0.05), the difference between the no-feedback group and the elaborated-feedback group was significant (*P* = 0.000 < 0.05), and the difference between the simple-feedback group and the elaborated-feedback group was not significant (*P* = 0.094 > 0.05). The total number of gazes significantly differed between the no-feedback group and the simple-feedback group (*P* = 0.000 < 0.05), between the no-feedback group and the elaborated-feedback group (*P* = 0.000 < 0.05), and between the simple-feedback group and the elaborated-feedback group (*P* = 0.695 > 0.05). The results of the one-way ANOVA for cognitive engagement are shown in Table 5.

**TABLE 5:** One-way analysis of variance (ANOVA) results for cognitive engagement.

Dependent variable	Sum of squares (between groups)	Mean square	F	Significance
Cognitive engagement subjective questionnaire data	50.383	25.192	2.716	0.072
Concentration	200.392	100.196	5.699	0.005
The total gaze time	2894.848	1447.424	14.003	0.000
The total number of gazes	56116.481	28058.241	14.497	0.000

#### 4.1.2 Behavioral engagement

Descriptive statistics of the subjective questionnaire data on behavioral engagement and hours of study were obtained, and the results are shown in Table 6.

**TABLE 6:** Results of the descriptive statistics of behavioral engagement.

Data sources	Dimension of measurement	Question feedback	Sample size	Average value	Standard deviation
Questionnaire	Behavioral engagement	No feedback	30	17.83	2.67
		Simple feedback	30	18.10	2.79
		Elaborated feedback	31	19.74	2.97
Brain wave instrument	Duration of study	No feedback	30	469.67	15.09
		Simple feedback	30	490.33	25.32
		Elaborated feedback	31	510.19	26.90

To further examine whether the subjective judgments of the behavioral engagement of the three groups were significantly different for the three types of feedback, a one-way ANOVA was conducted on the subjective judgments of behavioral engagement for the three groups. The results showed that the differences among the three groups of subjective behavioral engagement judgments reached the level of significance [F = 4.135 (2,88), *P* = 0.019 < 0.05]. *Post hoc* multiple comparisons of the subjective judgments of behavioral engagement using LSD revealed significant differences between the no-feedback group and the elaborated-feedback group (*P* = 0.010 < 0.05) and between the simple-feedback group and the elaborated-feedback group (*P* = 0.025 < 0.05) and revealed non-significant differences between the no-feedback group and the simple-feedback group (*P* = 0.714 > 0.05).

The type of feedback as the independent variable and the duration of study as the dependent variable were successively subjected to a variance chi-square test and one-way ANOVA. The results of the variance chi-square test showed that the variance in the three groups was not significant [F = 7.119 (2,88), *P* = 0.001 < 0.05]. The results of a one-way ANOVA showed that the difference between the three groups of study hours reached a significant level [F = 23.492 (2,88), *P* = 0.000 < 0.05]. Due to the heterogeneity of the variance in the length of study in the three groups, *post hoc* multiple comparisons of the length of study using the Tammany method revealed significant differences between the no-feedback group and the simple-feedback group (*P* = 0.001 < 0.05), between the no-feedback group and the elaborated-feedback group (*P* = 0.000 < 0.05), and between the simple-feedback group and the elaborated-feedback group (*P* = 0.013 < 0.05). The results of the one-way ANOVA for behavioral engagement are shown in Table 7.

**TABLE 7:** One-way analysis of variance (ANOVA) results for behavioral engagement.

Dependent variable	Sum of squares (between groups)	Mean square	F	Significance
Behavioral engagement subjective questionnaire data	65.484	32.742	4.135	0.019
The duration of study	25040.399	12520.200	23.492	0.000

#### 4.1.3 Emotional engagement

This study measured emotional engagement during panoramic video learning for three types of question feedback through emotional engagement questionnaire data and relaxation.

Descriptive statistics of the subjective questionnaire data on emotional engagement and relaxation were obtained, and the results are shown in Table 8.

**TABLE 8:** Results of the descriptive statistics of emotional engagement.

Data sources	Dimension of measurement	Question feedback	Sample size	Average value	Standard deviation
Questionnaire	Emotional engagement	No feedback	30	23.97	3.94
		Simple feedback	30	22.63	3.21
		Elaborated feedback	31	23.48	3.36
Brain wave instrument	Relaxation level	No feedback	30	51.02	5.05
		Simple feedback	30	56.55	6.40
		Elaborated feedback	31	55.18	5.38

To further examine whether there was a significant difference in the subjective judgments of emotional investment among the groups for the three types of feedback, a one-way ANOVA was conducted to analyze the subjective judgments of emotional investment among the three groups. The results showed that the differences in the subjective judgments of emotional investment among the three groups did not reach the level of significance [F = 1.105 (2,88), *P* = 0.336 > 0.05].

To further examine whether there was a significant difference among the relaxation levels of the groups of learners for the three types of feedback, a one-way ANOVA was conducted to analyze the relaxation levels of the three groups of learners. The results showed that the difference in learners’ relaxation levels among the three groups reached the level of significance [F = 7.842 (2,88), *P* = 0.001 < 0.05]. *Post hoc* multiple comparisons of learner relaxation using LSD revealed significant differences between the no-feedback group and the simple-feedback group (*P* = 0.000 < 0.05), between the no-feedback group and the elaborated-feedback group (*P* = 0.005 < 0.05), and between the simple-feedback group and the elaborated-feedback group (*P* = 0.344 > 0.05). The results of the one-way ANOVA for emotional engagement are shown in Table 9.

**TABLE 9:** One-way analysis of variance (ANOVA) results for emotional engagement.

Dependent variable	Sum of squares (between groups)	Mean square	F	Significance
Emotional engagement subjective questionnaire data	27.358	13.679	1.105	0.336
Relaxation	497.923	248.962	7.842	0.001

### 4.2 Academic performance

The descriptive statistics of the pre- and poststudy test scores are shown in Table 10.

**TABLE 10:** Descriptive statistics of the pre- and poststudy test scores.

Data sources	Dimension of measurement	Question feedback	Sample size	Average value	Standard deviation
Questionnaire	Pre-testing	No feedback	30	7.57	1.77
		Simple feedback	30	7.47	1.81
		Elaborated feedback	31	7.58	2.03
	Post-test	No feedback	30	8.77	2.33
		Simple feedback	30	10.03	2.04
		Elaborated feedback	31	11.23	1.84

To test whether the learners’ prior knowledge levels were similar, a one-way ANOVA was conducted with the pre-test scores of each group as the dependent variable, and the results showed that the differences between the total pre-test scores of the three groups did not reach a significant level [F (2, 88) = 0.033, *P* = 0.967 > 0.05]. The learners’ level of knowledge of “Deng Xiaoping, the Great Man” before they participated in the experiment tended to be similar, and prior knowledge and experience did not affect the results of the experiment.

The total post-test score, which was used as the dependent variable; the feedback format, which was used as the independent variable; and the total pre-test score, which was used as the covariate, were tested for between-subjects effects. The results showed that [F (2, 85) = 2.258, *P* = 0.111 > 0.05] the feedback format*total pre-test score, which indicates the total pre-test score of the learners in the groups with different feedback formats, did not significantly affect the total post-test score, and the relationship between the feedback format and post-test score can be tested with covariance analysis.

Based on this, a one-way covariance analysis of learners’ post-test academic performance across the feedback formats was conducted, with feedback format as the independent variable, post-test performance as the dependent variable, and the total pre-test performance score as the covariate. The results showed that the question feedback format significantly affected learners’ post-test performance [F (2, 87) = 21.460, *P* = 0.000 < 0.05].

Then, the question feedback type was further used as the independent variable, and the learners’ post-test scores were used as the dependent variable to analyze the extent to which the feedback form affected the post-test scores. After employing the LSD method to compare the groups with different feedback types, a *post-hoc* test based on the test revealed significant differences between the no-feedback group and the simple-feedback group (*P* = 0.001 < 0.05), between the no-feedback group and the elaborated-feedback group (*P* = 0.000 < 0.05), and between the simple-feedback group and the elaborated-feedback group (*P* = 0.005 < 0.05).

The *post hoc* comparison results of the effect of question feedback on learning engagement and academic performance are shown in Table 11.

**TABLE 11:** *Post hoc* comparisons of the effect of question feedback on learning engagement and academic performance.

Measure	(I) Feedback type	(J) Feedback type	Mean difference (I–J)	Std. error	Sig.	95% confidence interval for difference	
						**Lower bound**	**Upper bound**
Cognitive engagement questionnaire	No feedback	Simple feedback	−0.467	0.786	0.554	−2.03	1.10
	No feedback	Elaborated feedback	−1.752	0.780	0.027	−3.30	−0.20
	Simple feedback	Elaborated feedback	−1.285	0.780	0.103	−2.83	0.27
Concentration	No feedback	Simple feedback	−3.572	1.083	0.001	−5.723	−1.420
	No feedback	Elaborated feedback	−2.451	1.074	0.025	−4.585	−0.316
	Simple feedback	Elaborated feedback	1.121	1.074	0.299	−1.013	3.255
Total gaze time	No feedback	Simple feedback	−9.123	2.625	0.001	−14.3394	−3.906
	No feedback	Elaborated feedback	−13.537	2.604	0.000	−18.7114	−8.362
	Simple feedback	Elaborated feedback	−4.414	2.604	0.094	−9.588	0.761
Total number of gaze	No feedback	Simple feedback	−50.433	11.359	0.000	−73.007	−27.859
	No feedback	Elaborated feedback	−54.862	11.267	0.000	−77.253	−32.471
	Simple feedback	Elaborated feedback	−4.429	11.267	0.695	−26.820	17.962
Behavioral engagement questionnaire	No feedback	Simple feedback	−0.267	0.727	0.714	−1.71	1.18
	No feedback	Elaborated feedback	−1.909	0.721	0.010	−3.34	−0.48
	Simple feedback	Elaborated feedback	−1.642	0.721	0.025	−3.07	−0.21
Duration of study	No feedback	Simple feedback	−20.667	5.381	0.001	−33.99	−7.35
	No feedback	Elaborated feedback	−40.527	5.561	0.000	−54.29	−26.76
	Simple feedback	Elaborated feedback	−19.860	6.686	0.013	−36.29	−3.43
Emotional engagement questionnaire	Unable to perform LSD						
Relaxation	No feedback	Simple feedback	−5.528	1.455	0.000	−8.419	−2.636
	No feedback	Elaborated feedback	−4.156	1.443	0.005	−7.024	−1.288
	Simple feedback	Elaborated feedback	1.372	1.443	0.344	−1.496	4.239
Academic performance Questionnaire	No feedback	Simple feedback	−1.379	0.383	0.001	−2.139	−0.619
	No feedback	Elaborated feedback	−2.481	0.379	0.000	−3.235	−1.727
	Simple feedback	Elaborated feedback	−1.103	0.379	0.005	−1.857	−0.348

## 5 Discussion

### 5.1 The effect of the question feedback type on learning engagement

#### 5.1.1 The effect of the question feedback type on cognitive engagement

Combining the results of the subjective and objective measurement data revealed that the presence or absence of feedback information in panoramic videos embedded with questions significantly affects learners’ cognitive engagement, but there is no significant difference in the impact of simple feedback and elaborated feedback on cognitive engagement.

According to cognitive conflict theory, when learners receive feedback information and discover that new knowledge is inconsistent with existing knowledge, this can trigger cognitive conflicts among learners, thereby stimulating their learning motivation (Rahim et al., 2015). Cognitive engagement includes both psychological and cognitive factors. In terms of psychological factors, the learning motivation triggered by cognitive conflicts has a promoting effect and can make learners more willing to work hard to understand knowledge. In addition, as an important branch of social learning theory, Triadic reciprocal determinism was proposed by the famous American psychologist Bandura in the 1960s based on the Lewin model research (Chen and Li, 2015). From the perspective of Triadic reciprocal determinism, the process through which learners acquire knowledge is formed through the interaction of their individual factors, behaviors, and environmental factors. Individual factors refer to the intrinsic traits of learners, such as cognition and characteristics; behavior is the specific activity, action reflection, and external manifestation that learners can observe during the learning process; and environmental factors refer to the external environment that can influence learner behavior under the influence of individual factors. Embedding question feedback in panoramic videos creates an external environment for learners; learning engagement is the reflection of actions taken by learners during the learning process. When learners engage in internal self-regulation to learn knowledge, they receive feedback stimuli from the external environment, which can lead to monitoring and adjusting their cognitive processes. Learners learn through their own knowledge and experience. When the feedback information they receive conflicts with their existing cognition, their attention begins to increase, which will make certain adjustments to their cognitive process and make certain changes to the learning strategies that they use during the learning process. They exert psychological effort for the knowledge that they have learned, thereby affecting their cognitive engagement in the learning process. When a learner receives feedback that aligns with his existing cognition, he will consider the learning strategies and psychological efforts that he adopts appropriate and may monitor his cognitive process but will rarely make adjustments. Therefore, compared to no feedback, embedding question feedback in panoramic videos can improve learners’ cognitive engagement.

Previous studies have shown that learning engagement is influenced by the learning environment (Lawson and Lawson, 2013). Compared to a 2D environment, a 3D immersive VR environment can enhance learners’ interest and learning motivation (Huang et al., 2010). The learning motivation generated in a highly immersive environment such as panoramic videos promotes the improvement of cognitive engagement among learners. Because the level of precision in feedback information is weaker in stimulating learners than in the learning environment, there is no significant difference in the impact of simple feedback and elaborated feedback on cognitive engagement.

#### 5.1.2 The effect of the question feedback type on behavioral engagement

Combining the results of the subjective and objective measurement data showed that the presence or absence of feedback information in panoramic videos with embedded questions significantly affects learners’ behavioral engagement, and compared to simple feedback, elaborated feedback can better enhance learners’ behavioral engagement.

According to Triadic reciprocal determinism, in a panoramic video learning environment with embedded questions, learners receive feedback information after encountering and answering embedded questions. When the feedback information deviates from their expected expectations, learners adjust their learning strategies and invest more psychological effort, time, and energy, thus improving behavioral engagement. This provides a good explanation for why learners in the feedback group showed greater improvement in behavioral engagement than those in the no-feedback group showed.

When learners learn by using panoramic videos with embedded questions, they are unable to communicate with or receive timely feedback from the outside world. They can only solve problems independently and recall and connect them to their previous knowledge and experience. The emergence of feedback information allows learners to further actively explore, process, and construct the problems and difficulties that they encounter. According to the brainwave data, in terms of learning duration, the elaborated-feedback group had longer learning durations than the simple-feedback group had, indicating that learners in the elaborated-feedback group invested more time and energy, were more willing to spend time learning, and thus had improved behavioral engagement compared to the simple-feedback group. Wang et al. (2021) also used learning duration to reflect learners’ behavioral engagement and reported that learners spend more time learning in desktop VR environments than in traditional learning environments. Therefore, learners have better behavioral engagement in desktop VR environments. The reason for the inconsistency between the subjective and objective measures of variability of behavioral engagement in the no feedback group and the simple feedback group may be due to the fact that although the learners gave some time to pay attention to and learn from the simple feedback information, they spent more time in the question-answering activities, which resulted in the subjective perceptual judgment that they did not perceive that there was too much learning behavior and time invested in the simple feedback information.

#### 5.1.3 The effect of the question feedback type on emotional engagement

Combining the results of the subjective and objective measurement data revealed that the presence or absence of feedback information did not significantly affect emotional engagement. There was no significant difference between the simple-feedback group and the elaborated-feedback group.

The reason that the presence or absence of feedback information did not significantly affect emotional engagement may be that learners’ emotional learning states are stimulated when they learn using panoramic videos (Stavroulia et al., 2019), and the stimulus given may not have been sufficient to bring about a change in emotion, and learners learn in immersive learning environments with an overall higher emotional state, which leads to a non-significant difference in affective engagement, resulting in a non-significant difference in emotional engagement. Another reason why the fineness of the feedback information did not significantly affect emotional engagement may be that the learning materials were less difficult and met the learners’ expectations, which caused them to not put too much mental effort into the feedback information.

### 5.2 The effect of the question feedback type on academic performance

Research has shown that when learning from panoramic videos, learners who provide elaborated feedback tend to perform better in terms of academic performance.

When learners learn by using panoramic video teaching content without feedback, they may repeatedly think about whether they have answered questions correctly during the learning process; when learners learn using panoramic video teaching content with simple feedback, they may be disturbed by and doubtful because of their wrong answers. The provision of elaborated feedback can be an effective solution to these two situations. Feedback can help learners identify shortcomings in the learning process and make improvements, thereby enhancing academic performance (Lerdpornkulrat et al., 2019). With respect to the feedback principle, providing elaborated information to learners during a multimedia learning lesson aids in their knowledge construction and reduces cognitive processing demands (Mayer, 2014). The academic performance measured in this study refers to maintaining grades, and this conclusion is also in line with Mayer’s feedback principle.

### 5.3 Limitations and future directions

This study helps to expand the boundaries of feedback principles in multimedia learning and provides reference suggestions for the design and development of panoramic teaching videos. However, there are still some shortcomings in this study.

1. The study was conducted on undergraduate students, with an unbalanced gender ratio, and the study materials contained ideological and political content. Therefore, caution should be exercised in generalizing the findings to other groups or courses. It is worth exploring the study of research subjects of different ages and learning materials.
2. This study did not explore learners’ own characteristics as moderating variables, such as learners’ prior knowledge, learning styles, learners’ technology preferences, etc., and did not have a more detailed understanding of how these factors affect the relationship between question feedback types and learning engagement. Future research could explore the influence of learners’ own characteristics on learning engagement.
3. The learners’ emotional engagement in this study was measured by subjective questionnaires and relaxation scales, but it is worthwhile to further explore whether these measures reflect the learners’ actual emotional engagement.
4. This study was conducted in a laboratory environment, and further research is needed to investigate the effects of practical application in the classroom.
5. This study did not explore the effects of different multimedia interactive environments (e.g., VR, AR, MR, AI, etc.) on learning engagement. Future research could compare panoramic video with other interactive educational technologies to determine the specific strengths and weaknesses of this media.

## 6 Conclusion

This study explored the impact of question feedback on learning engagement in panoramic videos. The results showed that in panoramic video learning, providing feedback information can better enhance learners’ cognitive engagement, behavioral engagement, and academic performance than can providing only questions without feedback. Compared to simple feedback, elaborated feedback can better enhance learners’ cognitive engagement, behavioral engagement, and academic performance. Therefore, in the future, instructional designers can consider embedding questions and providing elaborated feedback when using panoramic video teaching to improve learners’ learning engagement and academic performance.

## Data Availability

The raw data supporting the conclusions of this article will be made available by the authors, without undue reservation.

## Appendix

**Appendix Table 1:** Panoramic video content and illustrations, questions with embedding and elaborated feedback.

Panoramic video content and illustrations	Questions with embedding	Elaborated feedback
Introduces the geographic location of Deng Xiaoping’s former residence, the main events of Deng Xiaoping’s childhood and adolescence.	1. Where is Deng Xiaoping’s former residence located? A Yilong County, Nanchong B Shunqing District, Nanchong C Xiexing Township, Guang’an City D Guang’an City Central District 2. Is Deng Xiaoping’s former residence just the place where Xiaoping lived as a child? ① Yes ② No	1. True/False, Deng Xiaoping’s former residence is located in Xiexing Town, Guang’an City 2. True/False, Deng Xiaoping’s former residence is the place where he lived as a child and teenager
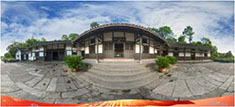
Introducing Comrade Deng Xiaoping’s famous quotes, major events in his youth after leaving Guang’an, including when he studied abroad, when he returned to China, and when he became Secretary General of the Central Committee of the Communist Party of China (CPC).	1. Deng Xiaoping took his Russian name while studying at Sun Yat-sen University in Moscow? A Dodorov B Lovoo C Blinsky D Darski 2. Chiang Kai-shek staged a counter-revolutionary coup d’état in Shanghai. In December of the same year, Deng Xiaoping traveled to Shanghai and became the secretary of the Central Committee of the Communist Party of China. ① Yes ② No	1. True/False, Deng Xiaoping’s Russian name was Dozorov when he studied at Sun Yat-sen University in Moscow 2. True/False, Chiang Kai-shek staged a counter-revolutionary coup in Shanghai. In July of the same year, Deng Xiaoping traveled to Wuhan as secretary of the CPC Central Committee, and in December, he traveled to Shanghai as secretary general of the CPC Central Committee
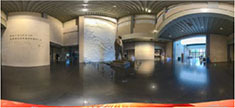
Introduces Deng Xiaoping’s major deeds for the establishment of a new China and the realization of the independence and liberation of the Chinese nation, including the uprisings he led or initiated, the founding of the Workers’ and Peasants’ Red Army, the Zunyi Conference, as well as the major deeds during the War of Resistance Against Japanese Aggression and the War of Liberation.	1. Deng Xiaoping was dismissed by the “left” leaders during his stay in the Left and Right River bases? ① Yes ② No 2. During the Liberation War, Deng Xiaoping and Liu Bocheng led their troops into the Dabie Mountains. ① Yes ② No	1. True/False, Deng Xiaoping was removed from his post by “left” leaders in the central base in Jiangxi because of his support for Mao Zedong’s correct line. 2. True/False, during the Liberation War, Deng Xiaoping and Liu Bocheng led an advance into the Dabie Mountains.
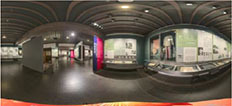
Introducing Deng Xiaoping’s main deeds after the country’s liberation and before the Cultural Revolution.	1. In March 1973, the Party Central Committee reinstated Deng Xiaoping to the position of? A premier of the State Council B Vice Premier of the State Council (PRC) C secretary-general of the Communist Party of China Central Committee D Chairman of the Military Commission (PRC) 2. Did Deng Xiaoping lose all his positions twice after the start of the Cultural Revolution in 1966? ① Yes ② No	1. True/False, Deng Xiaoping was removed from all his posts in the 1966 Cultural Revolution; in 1973, the Party Central Committee reinstated Deng Xiaoping as Vice Premier of the State Council 2. True/False, in 1966, Deng Xiaoping was removed from his post for the first time; in 1976, Deng Xiaoping was removed from his post for the second time
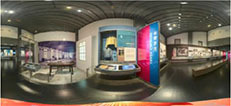
Introduction to the background and significance of the formation of Deng Xiaoping Theory and its contribution to the country’s reform and opening up, and the content and significance of the Basic Line at the Primary Stage of Socialism.	1. Which of the following ideas about Deng Xiaoping is not mentioned in the material? A Emancipate your mind and seek truth from facts B the four basic principles C The “three-step” strategy D One China, Two Systems 2. The Fifteenth Party Congress summarized the theory of building socialism with Chinese characteristics as Deng Xiaoping Theory, which was written into the Party Constitution as the Party’s guide to action. ① Yes ② No	1. True/False, the four basic principles are not mentioned in the materials 2. True/False, the 15th Party Congress summarized the theory of building socialism with Chinese characteristics as Deng Xiaoping Theory, which was written into the Party Constitution as the Party’s guide to action.
